# Neuropeptide Y Reduces Nasal Epithelial T2R Bitter Taste Receptor–Stimulated Nitric Oxide Production

**DOI:** 10.3390/nu13103392

**Published:** 2021-09-27

**Authors:** Ryan M. Carey, Nithin D. Adappa, James N. Palmer, Robert J. Lee

**Affiliations:** 1 Department of Otorhinolaryngology—Head and Neck Surgery, University of Pennsylvania Perelman School of Medicine, Philadelphia, PA 19104, USA; Ryan.Carey@pennmedicine.upenn.edu (R.M.C.); Nithin.Adappa@pennmedicine.upenn.edu (N.D.A.); james.palmer@pennmedicine.upenn.edu (J.N.P.); 2 Department of Physiology, University of Pennsylvania Perelman School of Medicine, Philadelphia, PA 19104, USA

**Keywords:** bitter taste receptors, neuropeptide Y, sinusitis, nitric oxide, cilia, innate immunity

## Abstract

Bitter taste receptors (T2Rs) are G-protein-coupled receptors (GPCRs) expressed on the tongue but also in various locations throughout the body, including on motile cilia within the upper and lower airways. Within the nasal airway, T2Rs detect secreted bacterial ligands and initiate bactericidal nitric oxide (NO) responses, which also increase ciliary beat frequency (CBF) and mucociliary clearance of pathogens. Various neuropeptides, including neuropeptide tyrosine (neuropeptide Y or NPY), control physiological processes in the airway including cytokine release, fluid secretion, and ciliary beating. NPY levels and/or density of NPYergic neurons may be increased in some sinonasal diseases. We hypothesized that NPY modulates cilia-localized T2R responses in nasal epithelia. Using primary sinonasal epithelial cells cultured at air–liquid interface (ALI), we demonstrate that NPY reduces CBF through NPY2R activation of protein kinase C (PKC) and attenuates responses to T2R14 agonist apigenin. We find that NPY does not alter T2R-induced calcium elevation but does reduce T2R-stimulated NO production via a PKC-dependent process. This study extends our understanding of how T2R responses are modulated within the inflammatory environment of sinonasal diseases, which may improve our ability to effectively treat these disorders.

## 1. Introduction

The nasal cavity is the first line of respiratory defense, with host–pathogen interactions occurring constantly. In most individuals, the sinonasal cavity remains free of infection due to exquisite epithelial innate defenses [1,2,3], including mucociliary clearance. Mucociliary clearance is complemented by the production of antimicrobial peptides (defensins, cathelicidin) and radicals (nitric oxide (NO), reactive oxygen species derived from hydrogen peroxide (H_2_O_2_)) [4]. Failure of these defenses can contribute to airway diseases such as chronic rhinosinusitis (CRS), which is a complex syndrome of sinonasal infection and/or inflammation affecting >35 million Americans [5,6,7,8,9,10,11] and accounting for one out of five adult antibiotic prescriptions [7,8,9,10].

Antibiotic-resistant organisms are increasingly found in patients with CRS and other airway diseases [12,13,14,15,16,17,18,19,20]. An attractive alternative therapeutic approach to antibiotics is to stimulate receptors that control endogenous immune responses, thus enhancing innate defenses without increasing pressures for single-agent resistance. One pathway that emerged over the last decade is the bitter taste receptor (T2R) pathway. Initially identified on the tongue, T2Rs are G-protein-coupled receptors (GPCRs) also expressed in other organs, including the bronchial and nasal motile cilia [4,21,22,23,24]. Several nasal cilia T2Rs detect bacterial products and activate local and rapid calcium-dependent nitric oxide (NO) synthase (NOS) activity, specifically via the eNOS isoform localized to the cilia and/or cilia base [25,26,27,28]. The NO produced increases mucociliary clearance and kills bacteria [22,29,30].

Isoforms T2R38 [22,31,32,33,34], T2R4 [35], and T2R14 [35] respond to acyl-homoserine lactone (AHL) quorum-sensing molecules produced by Gram-negative bacteria, including *Pseudomonas aeruginosa*. T2R4 and T2R14 also recognize quinolone quorum-sensing molecules’ *Pseudomonas* quinolone signal (PQS) and heptyl-hydroxy quinolone (HHQ), respectively [24,36]. T2R responses can also be evoked by other bacteria such as *Bacillus* species [37]. This supports T2Rs as a novel class of immune pattern recognition receptors, similar to toll-like receptors (TLRs).

The importance of T2Rs in nasal defense is supported by observations that patients who are homozygous for the AVI *TAS2R38* polymorphism, which renders T2R38 non-functional, have increased frequency of Gram-negative bacterial infections [22], higher levels of sinonasal bacteria [38,39,40], higher frequency of chronic rhinosinusitis [41,42,43,44], and worse outcomes after sinus surgery [45]. However, while T2Rs activate NO production in nasal cells, other receptors may inhibit it. It is important to understand how T2R responses are modulated within the inflammatory milieu of CRS and other airway diseases.

The focus of the current study was to determine how neuropeptide tyrosine (neuropeptide Y or NPY) might modulate T2R responses. Local sympathetic and parasympathetic neurons innervate nasal tissue [46,47] and release a variety of neuropeptides such as substance P (SubP), vasoactive intestinal peptide (VIP), and NPY, to control cell physiological processes such as fluid secretion, ciliary beating, and cytokine release. Parasympathetic neurons containing VIP and NPY [48,49,50,51,52,53,54,55,56,57] innervate the sinonasal cavity, including near submucosal glands of the turbinates [58,59,60] and at the pedicle of polyps [61,62]. Nasal NPYergic nerves may be increased in diseases such as allergic rhinitis [63,64] or irritative toxic rhinitis [65]. Activated macrophages can also produce NPY [66,67,68], possibly because NPY has antimicrobial effects [69,70,71] and/or regulates macrophage phagocytosis [72,73,74]. These neuropeptides activate GPCRs on epithelial and immune cells that regulate second messengers such as calcium, cAMP, and kinases. SubP and VIP stimulate nasal submucosal gland fluid secretion [75,76], and VIP increases nasal ciliary beating [77].

VIP and SubP receptors can activate cAMP and Ca2+ responses, respectively. VIP can increase ciliary beat frequency through cAMP [77]. NPY, however, decreases cilia beating through activation of NPYR2 receptors (our study [78] and others [79,80,81]). NPY also decreases fluid secretion from nasal submucosal gland cells via NPY1R receptor activation [75]. These negative effects of NPY are partly mediated by protein kinase C (PKC) activation [79,80,81,82]. Protein kinase C can phosphorylate eNOS at T495 [83,84,85] to reduce eNOS activity. Because T2Rs activate immune responses through eNOS, and NPY might inhibit eNOS function through activation of protein kinase C, we hypothesized that NPY may have negative effects on cilia-localized T2R responses. The increase in nasal NPYergic nerves during respiratory diseases described above may have a role in increasing susceptibility to infection by dampening T2R responses. The goal of this study was to better elucidate whether interactions occur between T2R and NPY signaling in nasal epithelial cells in vitro.

## 2. Materials and Methods

### 2.1. Reagents

Fluo-4-AM, DAF-FM-DA, DAF-2, anti-T2R38 (rabbit polyclonal; ab130503), and anti-beta-tubulin IV (mouse monoclonal; ab11315) were from Invitrogen (Grand Island, NY, USA). Anti-T2R14 (PA5-39710; rabbit polyclonal) was from Thermo Fisher Scientific (Waltham, MA, USA) and anti-eNOS (rabbit polyclonal; NB-300-605) was from Novus (Littleton, CO, USA). T2R14 antagonist 2-(4-fluorophenyl)-6- methoxychroman-4-one (4′- fluoro-6-methoxyflavanone) was purchased from VitaScreen, LLC (Champaign, IL, USA). Flavones, phorbol 12- myristate 13-acetate (PMA), ionomycin, L- and D-NG-nitroarginine methyl ester (L-NAME and D-NAME), U73122, and U73343 were from Cayman (Ann Arbor, MI, USA). Calphostin C, BIIE-0246, Gö6983, [Leu31,Pro34]-NPY, and NPY-(16–36) were from Tocris (Minneapolis, MN, USA). All other reagents were obtained from Sigma-Aldrich (St. Louis, MO, USA). Flavone stock solutions were made at 100 mM in DMSO. Final solutions used in experiments were made immediately before use and contained ≤0.1% DMSO.

### 2.2. Sinonasal Epithelial Air–Liquid Interface (ALI) Cultures

Patients undergoing sinonasal surgery were recruited from the University of Pennsylvania, Department of Otorhinolaryngology (IRB approval #800614). Patients were excluded if they had used anti-biologics (e.g., Xolair), oral corticosteroids, or antibiotics, within one month of surgery or had a history of immunodeficiency or systemic diseases (e.g., cystic fibrosis). All patients provided written informed consent in accordance with the code of federal regulation Title 45 CFR 46.116 of the United States Department of Health and Human Services. Experimental protocols were conducted in agreement with the research guidelines for use of residual clinical material outlined by the University of Pennsylvania.

Air–liquid interface (ALI) cultures were generated as previously described [24,86,87]. Briefly, enzymatically dissociated human sinonasal epithelial cells were then grown to confluence in proliferation medium (DMEM/Ham’s F-12 plus BEBM; Lonza, Walkersville, MD, USA) for one week. Following dissociation, cells were seeded onto a Transwell cell culture insert (0.4 µm pore size, transparent, 0.33 cm^2^ surface area; Corning, Corning, NY, USA) coated with fibronectin (Sigma), type I bovine collagen (Corning; Corning, NY, USA), and bovine serum albumin (Sigma) and in LHC basal medium (Invitrogen, Waltham, MA, USA). A One 10 cm culture dish (80–90% confluence) was used for two ALI plates (24-well format, each plate containing 12 transwells). After confluence, media was aspirated from the upper compartment. The basolateral medium was switched to differentiation medium (1:1 DMEM:BEBM) containing hydrocortisone (0.5 ng/mL), insulin (5 ng/mL), triiodothyronine (6.5 ng/mL), BPE (0.13 mg/mL), hEGF (0.5 ng/mL), epinephrine (5 g/mL), and transferrin (0.5 g/mL) from Lonza SingleQuot supplements plus 100 µg/mL streptomycin (ThermoFisher Scientific, Waltham, MA, USA), 0.1 nM retinoic acid (Sigma-Aldrich, St. Louis, MO, USA), 100 U/mL penicillin (ThermoFisher Scientific, Waltham, MA, USA), and 2% NuSerum (Corning, Corning, NY, USA).

### 2.3. Measurement of Ciliary Beat Frequency

Cultures were washed with PBS to remove media and maintained at ~28–30 °C. Dulbecco’s phosphate-buffered saline (PBS) (1.8 mM calcium) on the apical side and HEPES-buffered Hank’s balanced salt solution supplemented with 1× MEM vitamins and amino acids (ThermoFisher Scientific, Waltham, MA, USA) on the basolateral side. The Sisson–Ammons Video Analysis system (141) was used to measure whole-field ciliary beat frequency (CBF) as previously described [24,86,87]. A Leica (Wetzlar, Germany) DM-IL microscope (20× objective (0.8 NA)) with Leica IMC Hoffman modulation contrast was used for obtaining images at 100 frames/second. Each experiment was normalized to the baseline ciliary beat frequency at time = 0, as done previously [22,86,87]. Thus, the relative change in CBF was being reported.

### 2.4. Calcium and NO Imaging

Calcium and NO were imaged using primary sinonasal ALIs and Fluo-4 AM and DAF-FM, respectively. For calcium imaging, ALI cultures were loaded with 5 μM Fluo-4 AM apically for 90 min, washed, then incubated in the dark for 20 min. For NO imaging, cultures were loaded with 10 μM DAF-FM diacetate and 5 μM carboxy-PTIO apically for 90 min, washed, then incubated in the dark for 15 min. Imaging was performed using an Olympus IX-83 microscope (10 × 0.4 NA Plan Apo objective, Olympus Life Sciences, Tokyo, Japan) with a fluorescence xenon lamp (Sutter Lambda LS, Sutter Instruments, Novato, CA, USA), excitation and emission filter wheels (Sutter Instruments), and a 16-bit Hamamatsu Orca Flash 4.0 sCMOS camera. MetaFluor (Molecular Devices, Sunnyvale, CA, USA) was used to acquire images. A 470/40 nm excitation filter, 495 long-pass dichroic, and 525/40 nm emission filter were used (Chroma 49002-ET, Chroma Technologies, Rockingham, VT, USA). Each fluo-4 experiment was normalized to the baseline fluorescence at time = 0, as done previously [22,86,87] and is standard in the field with Fluo-4. Thus, normalized fluorescence was indicated by F/Fo. DAF-FM fluorescence is shown as raw increase after subtraction of baseline (time = 0), with care to keep loading and imaging parameters identical between all experiments.

For DAF-2 fluorescence measurement of the airway surface, liquid NO (1 µg/mL in PBS, 30 µL over 0.33 cm^2^) containing cell-impermeant NO-sensitive DAF-2 was used as described [22]. Solution was collected after 30 min, 25 µL was transferred to a glass-bottomed, black 96-well plate (CellVis, Mountain View, CA, USA), and fluorescence was read (485 nm excitation, 535 nm emission) in a fluorescence plate reader (Spark 10M, Tecan Männedorf, Switzerland).

### 2.5. Immunofluorescence

ALI cultures were fixed for 20 min in 4% formaldehyde at room temperature, followed by permeabilization and blocking for 1 h using PBS containing 5% normal donkey serum, 1% bovine serum albumin (BSA), 0.2% saponin, and 0.3% Triton X-100 at 4 °C. Primary antibody incubations (1:100 for anti-T2R antibodies, 1:250 for tubulin and eNOS antibodies) were performed at 4 °C overnight. Secondary antibody incubations with Alexa Fluor-labeled donkey anti-mouse or rabbit (1:1000) were performed at 4 °C for 2 h. Following primary and secondary antibody incubations, Transwell filters were cut from the plastic mounting ring with a razor blade and mounted onto glass slides with DAPI-containing Fluoroshield (Abcam, Cambridge, MA, USA). Zenon antibody labeling kits (Invitrogen/Molecular Probes/Thermo Scientific, Waltham, MA, USA) were used for direct labeling of primary antibodies. This was necessary to co-localize T2R38 and T2R14 as both antibodies were rabbit antibodies. Zenon labeling kits were used per the manufacturers’ instructions. An Olympus Fluoview confocal system with IX-81 microscope and 60× (1.4 NA) objective was used to take images of ALIs. The 60× (1.4 NA oil) objective on an inverted Olympus IX-83 microscope with spinning disk (DSU) running Metamorph (Molecular Devices, San Jose, CA, USA) was used for imaging isolated ciliated cells. Analysis of images was performed using Fluoview software, Metamorph, and/or FIJI [88].

### 2.6. Statistical Analyses

Analyses were performed using Prism (GraphPad, San Diego, CA, USA) with *p* < 0.05 considered to be statistically significant. One-way analysis of variance (ANOVA) was performed with appropriate post-tests. For comparisons to a control group, one-way ANOVA with Dunnett’s posttest was used. Bonferroni posttest was used for preselected pair-wise comparisons. Tukey–Kramer posttest was used for comparisons of all samples within a data set. All additional data analyses were performed in Excel. All figures are displayed with mean ± SD; one asterisk (*) indicates *p* < 0.05. Two asterisks (**) indicate *p* < 0.01.

## 3. Results

### 3.1. NPY Reduces CBF through NPY2R Activation of PKC

**Figure 1:**
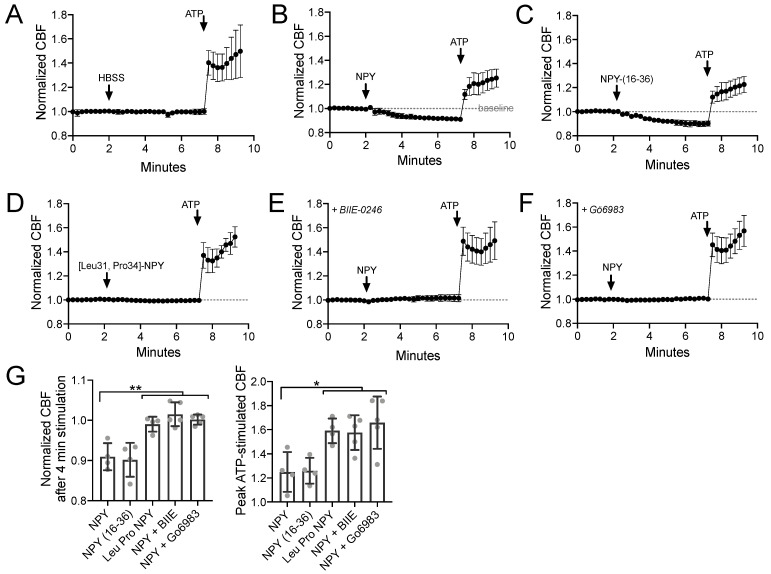
Neuropeptide Y (NPY) reduces baseline and adenosine trisphosphate (ATP)-stimulated ciliary beat frequency (CBF) through NPY receptor 2 (NPY2R) activation of protein kinase C (PKC). (**A**–**F**) Average traces of *n* = 4 experiments per condition, each experiment from a separate air liquid interface (ALI) culture from a separate chronic rhinosinusitis (CRS) patient. 100 µM ATP used as a control. (**G**) Bar graph showing raw data points from independent experiments and mean ± SD. Significance by one-way analysis of variance (ANOVA) with Dunnett’s posttest comparing all values to NPY alone; * *p* < 0.05 and ** *p* < 0.01. Other abbreviations: HBSS, Hank’s balanced slat solution; NPY-(16–36), NPY2R agonist; [Leu31, Pro34]-NPY, NPY1R agonist; BIIE-0246, NPY2R antagonist; Gö6983, PKC inhibitor.

Using primary sinonasal epithelial cells grown at an air–liquid interface (ALI), we observed that NPY (1 µM) reduced the baseline sinonasal ciliary beat frequency (CBF) (Figure 1A,B) in a manner that was mimicked by NPY2R agonist NPY-(16–36) (1 µM) but not NPY1R agonist [Leu31,Pro34]-NPY (1 µM) (Figure 1C,D). A high concentration of NPY (1 µM) was used in many experiments here as a saturating concentration to test effects of maximal stimulation of NPYRs. The effects of NPY were blocked by NPY2R antagonist BIIE-0246 (1 µM) or PKC inhibitor Gö6983 (1 µM) (Figure 1E,F). Results are summarized in Figure 1G. This confirms our previous observations that NPYR2 activation reduces CBF via PKC activation [78]. PKC is one of the major negative regulators of CBF [89].

### 3.2. T2R38, T2R14, and eNOS Have Different Patterns of Cilia Localization

**Figure 2:**
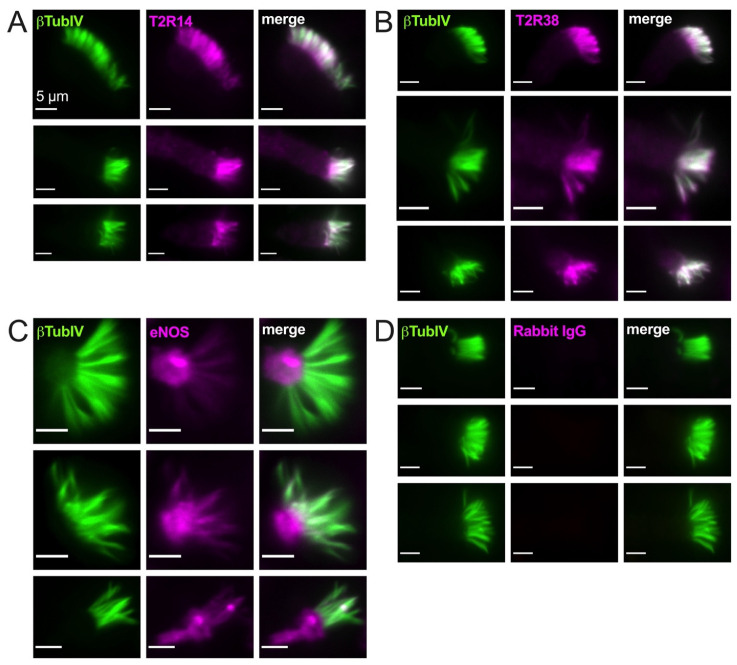
Bitter taste receptors T2R38 and T2R14 are localized to motile cilia while endothelial nitric oxide synthase (eNOS) is primarily localized at the base of cilia. Isolated ciliated cells brushed from middle turbinate tissue were stuck to glass using CellTak and fixed as described in the text and stained for β tubulin IV (βTubIV) and T2R14 (**A**), T2R38 (**B**), eNOS (**C**), or rabbit IgG ((**D**) isotype control for nonspecific antibody binding). All images were taken at identical microscope settings (exposure, LED power, binning, etc.) with 60× 1.4 NA oil objective.

As described above, PKC also negatively regulates eNOS, likely the major NOS isoform in healthy airway ciliated cells [90]. In isolated ciliated cells brushed from the middle turbinate tissue, we observed cilia localization of both T2R14 and T2R38 (Figure 2A,B) and some ciliary but mostly sub-ciliary apical localization of eNOS (Figure 2C,D). This confirms previous observations that eNOS is localized to the base of airway cilia [25,28].

### 3.3. NPY Attenuates CBF Response to T2R14 Agonists via NPY2R Receptors

**Figure 3:**
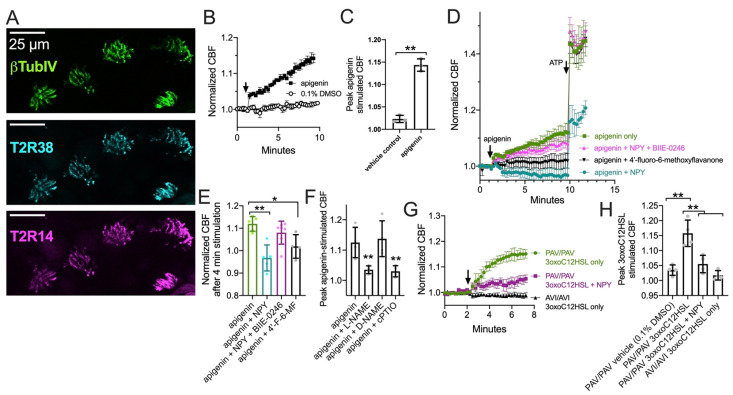
Neuropeptide Y (NPY) reduces ciliary beat frequency (CBF) increases in response to T2R14 agonist apigenin via NPY2R receptors. (**A**) Image of T2R14 and T2R38 in cilia from a primary nasal epithelial air-liquid interface (ALI) culture. (**B**) Ciliary response during stimulation with 100 µM apigenin or 0.1% dimethylsulfoxide (DMSO; vehicle control). (**C**) Bar graph (mean ± SD and individual data points; *n* = 3 independent experiments using cells from separate patients) of peak CBF after 8 min stimulation with apigenin or DMSO as in (**B**). (**D**) Ciliary responses during apigenin and subsequent ATP stimulation in the presence or absence of NPY, BIIE 0246 (NPY2R antagonist), and/or 4′-fluoro-6-methoxyflavanone (4′-F-6MF; T2R14 antagonist). Traces are average of 4–6 independent experiment using ALIs from different individual patients. (**E**) Individual data points and mean ± SD from experiments as in (**D**) showing CBF after 4 min stimulation with apigenin. (**F**) Peak apigenin-simulated CBF (mean ± SD and individual data points) from experiments as in (**D**) but with cultures treated with 10 µM L-NAME (NOS inhibitor), D-NAME (inactive analogue), or cPTIO (NO scavenger). (**G**) Ciliary responses during stimulation with 3oxoC12HSL (100 µM) in PAV/PAV (homozygous functional T2R38) or AVI/AVI (homozygous non-functional T2R38) genotyped cultures as well as subsequent ATP stimulation in the presence or absence of 10 nM NPY (lower concentration than used in (**D**–**F**). (**H**) Peak 3oxoC12HSL-simulated CBF (mean ± SD and individual data points) from experiments as in (**G**). Significance in (**C**) by Student’s *t*-test. Significance in (**E**,**F**) by one way ANOVA with Dunnett’s posttest comparing all values to apigenin only. Significance in (**H**) by one way ANOVA with Bonferroni posttest. * *p* < 0.05 and ** *p* < 0.01.

We tested T2R ciliary beat responses in nasal ALI cultures, which also express cilia-localized T2R14 and T2R38 (Figure 3A and [23,24]). The flavone apigenin is a T2R14 and T2R39 agonist that increases nasal epithelial cell CBF in a manner dependent on NO [23]. Apigenin (100 µM) increased CBF ~15% after 8 min, while vehicle control (0.1% DMSO) had minimal effect (Figure 3B,C). CBF increases in response to 100 µM apigenin were blocked by T2R14 and T2R39 antagonist [91] 4′-fluoro-6-methoxyflavanone (50 µM; Figure 3D,E). CBF responses to apigenin were inhibited with co-stimulation with 1 µM NPY (Figure 3D,E). This did not occur in the presence of 1 µM BIIE-0246 (Figure 3D,E). Together, these data suggest NPY negatively regulates T2R-mediated CBF responses, which are NO dependent as they are blocked by NOS inhibitor L-NAME (10 µM; 30 min pre-treatment) or NO scavenger cPTIO (10 µM; Figure 3F and [23,24]).

We also observed that *Pseudomonas aeruginosa* quorum-sensing molecule 3-oxo-dodecanoylhomoserine lactone (3oxoC12HSL; 100 µM), also a T2R agonist [22,92], increased CBF in a T2R38-dependent manner as previously reported [22]. CBF increased in cultures homozygous for the functional (PAV) TAS2R38 allele [93], while CBF did not increase in cultures homozygous for the non-functional (AVI) TAS2R38 allele (Figure 3G,H). We tested co-stimulation with a lower concentration of NPY (10 nM) than that used above. We found that NPY reduced the CBF response to 3oxoC12HSL in PAV/PAV cultures (Figure 3G,H). Thus, NPY can inhibit T2R-activated CBF increases.

### 3.4. NPY Does Not Alter T2R-Induced Calcium Elevation but Does Reduce T2R-Stimulated NO Production

**Figure 4:**
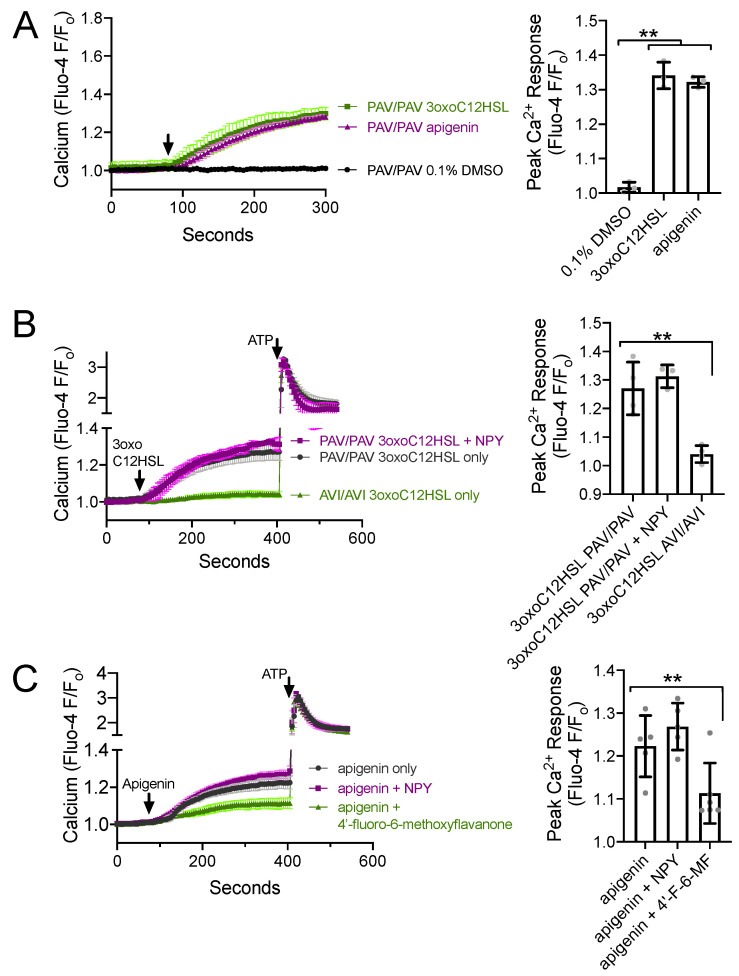
NPY does not alter T2R-induced calcium elevations. (**A**) Average calcium traces (**left**) and bar graph (**right**) showing mean ± SD of experiments in *TAS2R38* genotyped PAV/PAV (homozygous functional) ALIs stimulated with 100 µM 3oxoC12HSL, 100 µM apigenin, or 0.1% DMSO (vehicle control). Traces shown are mean from *n* = 3 experiments (each using a different ALI from a different patient). Significance on in bar graph determined by one-way ANOVA with Dunnett’s posttest comparing all values to 0.1% DMSO control; ** *p* < 0.01. (**B**) Average calcium traces (**left**) and bar graph (**right**) showing mean ± SD of experiments in *TAS2R38* genotyped PAV/PAV (homozygous functional) and AVI/AVI (homozygous nonfunctional) ALIs stimulated with 3oxoC12HSL ± NPY and subsequently with purinergic agonist ATP (100 µM). Traces shown are mean from *n* = 3–4 experiments (each using a different ALI from a different patient). Significance in bar graph determined by one-way ANOVA with Dunnett’s posttest comparing all values to 3oxoC12HSL in PAV/PAV cultures (first bar); ** *p* < 0.01. (**C**) Average calcium traces (**left**) and bar graph (**right**) showing mean ± SD of experiments in ALIs stimulated with T2R14 agonist apigenin ± NPY or 4′-fluoro-6-methoxyflavanone (T2R14 antagonist). Significance in bar graph determined by one-way ANOVA with Dunnett’s posttest comparing all values to apigenin only (first bar); ** *p* < 0.01. *TAS2R38* PAV/AVI cultures were used here as *TAS2R38* does not affect responses to apigenin (previously shown in [23]).

Because T2R-stimulated NO production via eNOS is driven by calcium signaling [22,23,24], one potential explanation for the above observations is that NPY reduces the T2R-stimulated calcium response. To test this, we imaged T2R-induced calcium responses in ALIs loaded with fluorescent calcium-sensitive dye Fluo-4. Both *P. aeruginosa* [22] and apigenin [23] activate calcium responses in primary sinonasal cells grown at ALI. We confirmed that 100 µM 3oxoC12HSL and 100 µM apigenin activated calcium responses in ALIs derived from patients homozygous for functional (PAV) T2R38, while the 0.1% DMSO vehicle control solution had no effect on calcium (Figure 4A). As previously reported [22], we found that 3oxoC12HSL activated calcium responses in PAV/PAV ALIs but not in ALIs derived from patients homozygous for non-functional (AVI) T2R38 (Figure 4B). The calcium response in PAV/PAV ALIs was not reduced in the presence of 1 µM NPY (Figure 4B), which is likely a saturating, supraphysiological, maximally activating concentration of NPY. Likewise, the calcium response to T2R14 agonist apigenin (100 µM) was not reduced by 1 µM NPY but was reduced by T2R14 antagonist 4′-fluoro-6-methoxyflavanone (50 µM; Figure 4C). Thus, the reduced CBF response during NPY co-stimulation was not likely due to reduced calcium signaling.

**Figure 5:**
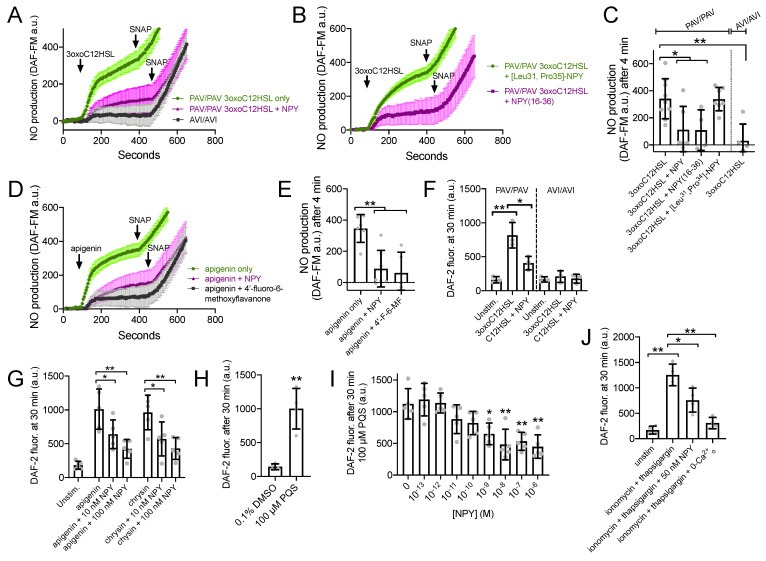
NPY reduces T2R-stimulated NO production. (**A**,**B**) DAF-FM traces (average of 6–9 experiments using ALIs from 2–3 different patients) showing NO production in response to 100 µM 3oxoC12HSL ± 1 µM NPY, 1 µM [Leu31,Pro35]-NPY (NPY1R agonist) or 1 µM NPY(16–36) (NPY2R agonist). 10 µM SNAP (nonspecific NO donor) used at the end of each experiment as a positive control. (**C**) Bar graph (mean ± SD) showing 3oxoC12HSL DAF-FM responses from experiments as in (**A**). Significance by one-way ANOVA with Dunnett’s posttest comparing all values to 3oxoC12HSL alone in PAV/PAV cultures (first bar); * *p* < 0.05 and ** *p* < 0.01. (**D**) Experiments as in (**A**,**B**) but with T2R14 agonist apigenin ±1 µM NPY or 50 µM 4′-fluoro-6-methoxyflavanone. (**E**) Bar graph (mean ± SD) showing apigenin DAF-FM responses from experiments in (**D**). Significance by one way ANOVA with Dunnett’s posttest comparing all values to apigenin only (first bar). (**F**) Bar graph (mean ± SD) of DAF-2 fluorescence in apical surface liquid of ALI culture stimulated as indicated by 100 µM 3oxoC12HSL ± 100 nM NPY in PAV/PAV or AVI/AVI cultures as indicated. Note lower concentration of NPY was used here vs. 1 µM in (**B**–**E**). Significance by one-way ANOVA with Dunnett’s posttest comparing all values to unstimulated PAV/PAV cultures (first bar); * *p* < 0.05 and ** *p* < 0.01. (**G**) Bar graph (mean ± SD) of data from experiments as in (**F**) but performed with T2R14 agonists apigenin and chrysin (100 µM each) ± 10 nM or 100 nM NPY (lower, more physiological concentrations). Significance by one-way ANOVA with Bonferroni posttest; * *p* < 0.05 and ** *p* < 0.01. (**H**) *Pseudomonas* quinolone signal (PQS; 100 µM) stimulation resulted in increased apical DAF-2 fluorescence over vehicle control (0.1% DMSO; *n* = 4 experiments per condition using ALIs from separate patients). Significance by Student’s *t*-test; ** *p* < 0.01. Bar graph shows mean ± SD. (**I**) Experiments were performed as in (**H**) with 100 µM PQS ± a dose response of NPY (*n* = 5 experiments per condition, each using an ALI from a different patient). Significance by one way ANOVA with Dunnett’s posttest comparing all values to PQS with no NPY (0 NPY; first bar); * *p* < 0.05, ** *p* < 0.01. (**J**) Experiments as in (**F**,**G**) but with ionomycin and thaspigargin and 50 nM NPY. Last bar shows inhibition of the ionomycin and thapsigargin response in extracellular calcium-free media (0-Ca^2+^_o_), with no added calcium plus 2 mM EGTA to chelate trace calcium. Significance by one-way ANOVA with Bonferroni posttest; * *p* < 0.05 and ** *p* < 0.01. Bar graph shows mean ± SD.

To directly measure T2R-induced NO production, we imaged cultures loaded with the NO-sensitive dye DAF-FM. DAF-FM fluorescence increased in response to 100 µM 3oxoC12HSL in ALIs from PAV/PAV but not in AVI/AVI patients (Figure 5A,C). Non-specific NO donor SNAP (10 µM) was added as a control at the end of each experiment. NO production in PAV/PAV ALIs was reduced during co-stimulation with 1 µM NPY (Figure 5A,C). NPY2R agonist NPY-(16–36) also reduced 3oxoC12HSL NO production in PAV/PAV ALIs, while NPY1R agonist [Leu31, Pro35]-NPY had no effect (Figure 5B,C). Similarly, apigenin-induced DAF-FM/NO responses were reduced with NPY or T2R14 antagonist 4′-fluoro-6-methoxyflavanone (Figure 5D,E).

We tested NO release into the airway surface liquid by overlaying ALIs with impermeant dye DAF-2 as previously done [22]. DAF-2 fluorescence increased, signaling NO production, in the presence of 100 µM 3oxoC12HSL in *TAS2R38* PAV/PAV (functional T2R38) cultures, and this was reduced by 100 nM NPY (Figure 5F). No increase in DAF-2 fluorescence was observed with *TAS2R38* AVI/AVI (non-functional T2R38) cultures, showing this response was dependent on T2R38 (Figure 5F). T2R14 agonists apigenin and chrysin also increased DAF-2 fluorescence, and this was likewise reduced by 10–100 nM NPY (Figure 5G). T2R4 agonist *Pseudomonas* quinolone signal (PQS; [24]) also increased apical DAF-2 fluorescence over vehicle (0.1% DMSO; Figure 5H). A dose response of NPY co-stimulation in this assay (from 10^−13^ to 10^−6^ M or 0.1 pM to 1 µM), revealed statistically significant inhibition of DAF-2 fluorescence increase with as low as 1 nM NPY.

We also found that 50 nM of NPY was sufficient to reduce the NO produced during application of calcium ionophore ionomycin combined with calcium ATPase inhibitor thapsigargin (1 µg/mL each; Figure 5J). Because these compounds will elevate calcium independent of GPCR signaling, the inhibition of NO production here supports that the inhibitory effect of NPY was likely downstream of calcium.

### 3.5. NPY Effects on T2R-Mediated NO and CBF Responses Are Dependent on PKC

**Figure 6:**
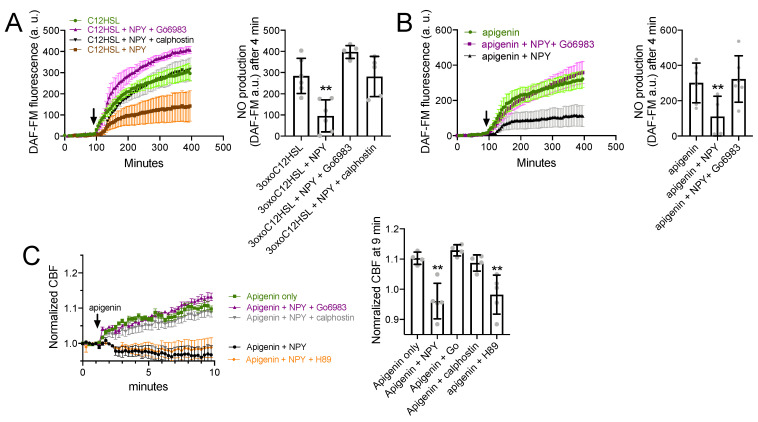
NPY effects on T2R NO and CBF responses are dependent on PKC. (**A**) DAF-FM responses (*n* = 5 experiments per condition, each experiment using an ALI from a different TAS2R38 PAV/PAV patient) showing responses to 100 µM 3oxoC12HSL ± 1 µM NPY ± PKC inhibitors 1 µM Gö6983 or 1 µM calphostin C. Significance in bar graph determined by one-way ANOVA with Dunnett’s posttest comparing all values to 3oxoC12HSL alone (first bar); ** *p* < 0.01. (**B**) Experiments as in A but in TAS2R38 PAV/AVI cultures stimulated with apigenin (100 µM) ± 1 µM NPY ± 1 µM Gö6983. Significance in bar graph determined by one-way ANOVA with Dunnett’s posttest comparing all values to apigenin alone (first bar); ** *p* < 0.01. (**C**) CBF responses to 100 µM apigenin ± 1 µM NPY ± 1 µM Gö6983, calphostin C, or H89 (PKA inhibitor used as a control). Significance in bar graph determined by one-way ANOVA with Dunnett’s posttest comparing all values to apigenin alone (first bar); ** *p* < 0.01. All bar graphs are mean ± SD.

Data above show that NPY stimulation reduces T2R-mediated NO responses without a change in upstream calcium signaling. To find whether this was due to protein kinase C activation, We imaged DAF-FM-loaded PAV/PAV ALIs stimulated with 3oxoC12HSL (100 µM). Stimulated NO production was reduced in the presence of 1 µM NPY but was not reduced in the presence of NPY plus either of two PKC inhibitors, 1 µM Gö6983 or 1 µM calphostin C (Figure 6A). Apigenin (100 µM)-induced NO production was likewise inhibited by 1 µM NPY but restored in the presence of 1 µM Gö6983 (Figure 6B). Apigenin-induced CBF increase was likewise reduced by 1 µM NPY but unaffected in the presence of 1 µM Go6983 or calphostin C (Figure 6C).

## 4. Discussion

We show an inhibitory effect of NPY on T2R-mediated NO production and CBF responses. This likely occurs through activation of NPY2R receptors and subsequent PKC activation. We previously showed that activation of PKC downstream of fungal aflatoxins can reduce T2R-mediated NO production [78]. We hypothesize this is because of eNOS T495 phosphorylation by PKC, which impairs calcium-bound calmodulin binding to eNOS [94]. NO increases cilia beating and mucociliary clearance through activation of guanylyl cyclase and protein kinase G [89]. Thus, elevated NPY in CRS or asthma may impact cilia function, perhaps contributing to susceptibility to infection by reducing mucociliary clearance. While T2R-induced changes in CBF are somewhat small (10–20%), the relationship between CBF and mucociliary clearance rate (as measured in fluorescent particle transport assays) is not linear. We previously showed that these T2R-induced 10–20% changes in CBF equate to ~50–80% increases in mucociliary transport [22,29]. Thus, the blunting of T2R responses by NPY likely has even greater effects on mucociliary clearance than the changes in CBF observed here.

We observe effects of NPY on T2R-induced NO responses as low as 1 nM. The pKd of recombinant NPY2R vs I125-NPY was reported to be ~10.17 (~0.068 nM) [95] and EC_50_ of ~0.3 nM in a heterologous expression system [96]. The apparent EC_50_ for NPY is likely higher than the binding of NPY to its receptor due to downstream nonlinearity. Thus, while many experiments in this study and other studies (e.g., [97,98]) test high saturating concentrations of NPY (e.g., 100 nM–1 µM) to maximally activate receptors, we also see that lower, likely more physiological concentrations are also sufficient to reduce T2R responses. Thus, while in vivo and other further experimentation is needed to understand the potential role of this mechanism in sinonasal disease, our data suggest that the inhibition of T2Rs reported here may be activated by physiologically relevant levels of NPY that activate NPY2R. This is particularly true in cells in the nose in close proximity to NPYergic fibers. Local release of neurotransmitters in the airway could generate high nM to µM concentrations within close proximity to the neuron, albeit likely for short periods of time [99].

Notably, we show that NPY blunts responses to *P. aeruginosa* 3oxoC12HSL and PQS, detected largely by T2R38 [22] and T2R4 [24], respectively. We also show blunted responses to apigenin, which we previously concluded was mediated by T2R14 [23]. However, apigenin and other types of flavones also activate T2R39 [100,101,102]. The 4′- fluoro-6-methoxyflavanone antagonist used here also blocks T2R39 [91]. We have not detected T2R39 in differentiated nasal cilia [23,24], but it is expressed in bronchial cilia [21]. We thus cannot fully rule out a potential role for T2R39 in the apigenin response. Nonetheless, this does not change any of the conclusions of this paper. NPY still blunts the T2R-mediated response to apigenin, whether through T2R14 alone or a combination of T2R14 and T2R39. We did not test agonists for T2R16, which is also in nasal cilia. As we have shown that all of these T2Rs signal identically [4,24], we believe it is likely that T2R16 responses are likewise reduced by NPY.

Beyond ciliary beating, NO is also important to airway immunity because it directly kills or inactivates pathogens. NO damages the DNA and cell walls of bacteria [103,104,105,106,107]. Replication of many respiratory viruses is also NO sensitive, including influenza, parainfluenza, rhinovirus [108], and SARS-COV1 and 2 [109,110,111,112]. Reduced NO output by the nasal epithelium with NPY elevations may contribute to susceptibility to both bacterial and viral infection. Plant flavonoids such as the flavones apigenin and chrysin are attractive molecules to target nasal T2Rs because they also have intrinsic antibacterial properties [23,113]. However, there ability to activate T2R14 may be limited in patients with elevated NPY levels.

As noted above, NPY may be elevated in asthma, which does carry a greater risk of respiratory infection [114,115,116]. As T2Rs are also expressed in bronchial cilia [21], impairment of T2R function by NPY in asthma may also contribute to innate immune dysfunction in this disease. Future studies of bronchial epithelial cells are needed to clarify the role of NO in the bronchial epithelial T2R CBF response. PKC inhibitors have been suggested as therapeutic modalities for several types of chronic inflammatory diseases [117] and might be useful in chronic inflammatory airway diseases where NPY is elevated.

In mice, NPY may be critical in type 2 inflammatory responses in the airways [118,119,120]. However, while some studies have demonstrated elevated human sinonasal NPY expression in different types of sinonasal diseases [62,63,64,65,121,122], a greater study of changes in NPY levels and localization in the nose in specific CRS patient subsets is needed to better understand whether and how NPY fits into the context of CRS pathophysiology. In particular, future measurement of NPY levels, localization of NPY production, and elucidation of potential changes in normal vs. diseased inflamed sinonasal tissue are all needed. While sensory neurons and macrophages are a potential source of NPY production, other immune cells such as neutrophils or eosinophils might also produce NPY and alter epithelial responses to bitter bacterial or fungal molecules. The T2R-to-eNOS signaling pathway also functions in macrophages, where it regulates phagocytosis [123], and macrophage-produced NPY may feed back onto this pathway in an autocrine signaling loop. An overall better understanding of neuropeptides in both upper and lower airway inflammation is needed to understand whether targeting these pathways can reduce inflammation and/or boost innate immunity.

We previously showed that NPY is a negative regulator of nasal turbinate submucosal gland secretion via the activation of NPY1R receptors [75]. Nasal and bronchial submucosal glands are important for the secretion of not only fluid for airway surface liquid and mucus but also antimicrobial peptides [76,124]. Reducing both glandular antimicrobial peptide secretion and antimicrobial NO production suggests that NPY has multiple negative effects on sinonasal innate immunity. However, future studies are needed to clarify what relevance the in vitro observations here have to in vivo sinonasal pathophysiology. It may be that the function of NPY as a “brake” on ciliated cell NO production and CBF is somehow beneficial. Regardless, the studies here demonstrate that NPY is a potent regulator of epithelial T2R NO, warranting further investigation into the role of NPY in sinonasal innate immunity.

NPYRs can also be activated by pancreatic polypeptide (PP) and peptide YY (PYY). While PYY is generally thought to be only produced by PP cells (also known as gamma or F cells) in pancreatic islets, some PYY is found in gastrointestinal epithelial neurons [60]. To our knowledge, studies of PP and PYY expression in the nasal cavity are extremely limited [125]. Future studies must explore these other NPY family polypeptides. Future studies are also needed to determine whether and how other neuropeptides sch as substance P and VIP may also affect T2R signaling in nasal epithelial ciliated cells.

## Data Availability

All data supporting the results are contained within the article figures. Raw numerical values used to generate the bar graphs or traces are available upon request to corresponding author.
